# Factors Associated With Patient Satisfaction in Outpatient Department of Suva Sub-divisional Health Center, Fiji, 2018: A Mixed Method Study

**DOI:** 10.3389/fpubh.2019.00183

**Published:** 2019-07-02

**Authors:** Swastika Chandra, Paul Ward, Masoud Mohammadnezhad

**Affiliations:** ^1^Department of Public Health, School of Public Health and Primary Care, Fiji National University, Suva, Fiji; ^2^Department of Public Health, School of Health Sciences, Flinders University, Adelaide, SA, Australia

**Keywords:** patient satisfaction, determinants, trust, communication, mixed method study, Fiji

## Abstract

**Background:** With evolving health care industry toward patient centered orientation, inputs from the patients' perspective is valuable. Improved patient satisfaction is associated with increased levels of adherence to treatment processes and recommended prevention, and improved health outcomes. Hence, this study was conducted to assess the current level of patient satisfaction and explore its determinants in the Suva Subdivision health centers, Fiji, 2018.

**Methods:** This was a mixed method cross-sectional study employing both quantitative and qualitative designs. A random sample of 410 participants attending the outpatient services completed the self-administered structured questionnaire. The questionnaire focused on socio-demographic features, waiting time, doctors' communication, and patient trust. Data from 375 questionnaires (response rate of 91%) was analyzed in SPSS where descriptive analysis and univariate and multivariate logistic regression was done at 0.05 level of significance and 95% confidence interval to find the determinants of patient satisfaction. From these 375 participants, 20 participants were purposefully selected for audio recorded interview guided by a semi-structured questionnaire and data was analyzed using thematic analysis.

**Results:** The majority of the patients were generally fully satisfied with their consultation (69.3%). Univariate logistic regression showed that age, gender, education level, waiting time, doctors' communication behavior, and patient trust level were significantly associated with patient satisfaction independently. After controlling for all the variables, gender, number of visits, waiting time, and patient trust were significantly associated with trust. Those who had full trust in the doctors, were more likely to be fully satisfied with their consultation (aOR of 18; *p* = 0.0001) and those who got seen within 1 h, were more likely to be satisfied with their consultation (aOR of 3.3; *p* = 0.0001). Though, the patients voiced that getting a satisfying consultation was worth the wait. The doctors' attitude and way of communication also made a difference to the patient's level of satisfaction.

**Conclusions:** This study showed that patient satisfaction is positively associated with patient trust, doctors' interpersonal skills and communication behavior and negatively associated with waiting time. Hence, doctors upgrading their communication skills and health service managers strategizing ways to improve waiting time can contribute to better patient trust and thus lead to better patient satisfaction and positively influence health outcomes.

## Background

The health care system has evolved over time with a shift from being a traditional concept of noble profession toward a customer-oriented service industry (1). Contributing to this shift in health care system includes factors such as the availability of information through internet, higher expectations of patients, health insurance schemes, and advancement in medical technology (1). This has resulted in a challenge for the healthcare industry in delivering high quality of health care services; safe, equitable, evidence based, timely, efficient, and patient centered services (2). Patient satisfaction is, “a measure of the extent to which a patient is content with the health care which they received from their health care provider” (3). It measures the perceived level of care quality and acts as a means of feedback for health care providers by giving healthcare providers valuable insights into various aspects of health care, such as the effectiveness of their care and their level of understanding (4).

Patient satisfaction surveys has been used as a meaningful and essential tool for identifying gaps and developing effective strategies for quality improvements in health care industry (5). Given the change in the health care industry toward patient centered orientation, inputs from patients' perspective is valuable (1, 6). Studies have shown that some of the factors associated with patient satisfaction include patient age and gender, continuity of care, waiting time, communication, and patient trust (7–16). Patients' trust in their doctors and effective communication between doctor and patients has been shown to have positive effects on health outcomes as well (17, 18). Trust has been shown to have a positive impact on patients, such as patient's adherence to medication, patient satisfaction, and a better indicator of follow up treatment (18–21). Patients with higher trust in their physician usually have more beneficial health behaviors, less symptoms, higher quality of life and were more satisfied with the treatment (17, 22–25). Though, these patients may not be necessarily experiencing objectively better clinical outcomes as a meta-analysis study showed that was moderate correlation between patient trust and a subjective review of clinical outcomes (17). Therefore, indicating that a trusting patient-provider relationship makes patients believe they are receiving better care. Patient trust, proper doctor-patient communication and patient satisfaction are interlinked (15, 16). Hence, patient satisfaction can be considered as an indirect measure of self-rated subjective health outcomes and to some extent objective health outcomes.

Price et al. in their literature review showed that improved patient satisfaction was associated with increased levels of adherence to treatment processes and recommended prevention, improved clinical outcomes, better patient safety within hospitals and less health care utilization (22). Biglu et al. carried out a cross-sectional descriptive study among the patients referred to 8 specialized clinics of Tabriz University of Medical Sciences in Iran. The study showed strong correlation between the communication skills of doctors and patient satisfaction. The study concluded that doctors should be having regular workshops to improve their communication skills (16). Trust, interaction and empathy have also been shown to have a positive influence on patient satisfaction. Therefore, conducting patient satisfaction surveys using standard protocols and appropriate methods are appropriate complements for clinical process and outcome measures in health care facilities. These experiences of the patients gathered from a patient's report carry a significant weight.

Despite the increasing importance and research on patient trust, communication and patient satisfaction, there are limited studies done in Pacific Island Countries (PICs) on these subjects. This study was conducted with the purpose to assess the current perceived level of patient satisfaction in resource strained PIC, with Fiji being a representive and the factors associated with patient satisfaction. This study also tries to assess how strongly trust and communication are associated with patient satisfaction.

## Materials and Methods

### Study Setting and Design

This was a cross-sectional mixed method study employing both quantitative and qualitative designs using the concept of methodological triangulation (26). The quantitative approach assisted in providing an accurate method to measure patient perceived doctor's communication behavior, trust, and patient satisfaction. Qualitative approach with in-depth interview with the patients was carried out to gather in depth information on this topic and assisted in better understanding patient's perspective on the topic and the subtle variations in the patient's experiences (27–29). Study was conducted in the outpatient department of randomly selected health centers in the Suva Subdivision.

### Sampling and Sample Size

For the quantitative approach, the estimated sample size was calculated using 5% margin error and 95% confidence interval and 50% response distribution. With the use of these figures, the estimated sample size comes to 377. In view of non-response of 10%, the sample size came to 410 participants. To get the total of 410 participants, the number of patients selected from the three-individual health centers depended on the health centre's usual patient load. Participants were selected using systematic random sampling. The patients waiting to be consulted by the doctors in the outpatient department were approached. Every third person waiting to be seen was approached and those who were interested, and after reading the information sheet, those who were still willing to participate and meet the inclusion and exclusion criteria were selected to participate in the studies.

### Inclusion and Exclusion Criteria

Those who were over 18 years, any gender, any ethnic background, self-identified Fijians, and understood either English, Hindi, or Fijian were included in the study, while those not willing to participate or had some form of cognitive disorder were excluded from the study (This included conditions such as mental retardation, stroke, or uncontrolled psychiatric condition. The recognition of the cases was from self-recognition of obvious cases and some from the history as relayed by the relative or caregiver who accompanied the patient). The selected participants' details were listed down, and this list was used to select participants for the qualitative approach. This list was divided into 6 strata; male, female, I-Taukei, Indo-Fijian, 18–50 years, and more the 50 years of age. From these sub-groups, convenience sampling was used to select the participants for the in-depth interview. The total participants were 20; inclusive of 5 participants representing each sub-group.

### Data Collection Tool

A structured questionnaire with close-ended questions was used for the quantitative design. The statements in this questionnaire have been already used in previous studies concerning trust, communication skills, and patient satisfaction (30–34). Questions and statements from these already tested questionnaires were used to make the questionnaire for this study; trust in physician scale, communication assessment tool and patient satisfaction surveys. The statements/questions used had high internal reliability with a Cronbach's score of 0.7 or more (30–34). Content validity was verified by two experts. Face validity was done by giving the printed questionnaires to 10 patients in the outpatient department (these patients were not included in the final study) to assess whether it is legible, clear, simple, and easy to understand. Most of the patients understood the statements and found it easy to fill and returned within 10 min, thus, there was no changes made to it. The variables from this questionnaire used in analysis included patient satisfaction, age, sex, ethnicity, educational level, and employment status, number of visits to the health center, waiting time for the consultation, communication behavior of doctors and patients trust in doctors.

The questionnaire contained 4 sections; section 1 contained the socio-demographic information of the participant, section 2 contained 8 close-ended questions which were related to communication skills of the doctors, section 3 contained 10 close-ended questions which were related to patient trust in doctors, and section 4 contained 4 close-ended questions assessing patient satisfaction. Likert 5-point scoring scale was used for answering the questions; strongly agree, agree, not sure, disagree and strongly disagree. Strongly agree is allocated 5 points and regresses down to 1 point for strongly disagree for positive statement such as, “My doctor gave me opportunity to ask questions.” For negative statement such as, “I would prefer to get a second opinion from another doctor,” strongly agree is allocated 1 point and strongly disagree is allocated 5 points. The questionnaire was translated into two other languages; Fijian and Hindi using bilingual translators. Theses translated version were then translated back from Hindi and Fijian to English by different translators to ensure the contents of the original questionnaire matches the translated version (34).

Though, the questionnaire was not tested for reliability before being used for the study, the reliability was tested, and the Cronbach alpha was calculated using this study sample (375 questionnaires). For the whole questionnaire, the Cronbach alpha score for the total of 22 items was 0.91. For section 2, with a total of 8 items, the Cronbach alpha score was 0.76, for section 3 with 10 items was 0.759, and section 4 with 4 items was 0.81. Standardized open-ended audio recorded interview was conducted which was 30–40 min long per interview. The interview was guided by a semi-structured questionnaire that used 8 open-ended questions which focused on participants opinion and feelings about trust in doctors, reasoning behind their trust and satisfaction with the consultation.

### Data Analysis

The quantitative raw data from the questionnaire was entered into excel and after data cleaning, was transferred into IBM SPSS, version 21. Descriptive analysis (mean, standard deviation, and frequency) was done for the socio demographic variables, waiting time, communication behavior, trust, and patient satisfaction. For inferential analysis, logistic regression was carried out as the variables did not have a linear relationship. Hence, the variables, patient satisfaction, communication behavior, trust, and age were converted into categorical variables whereas the others were already categorical variables. All the statements were scored depending on the participants' choice for sections 2–4.

The maximum score of 5 points and minimum score of 1 point. Communication behavior was categorized as “Good communication behavior (32–40 points),” “Fair communication behavior (17–31 points),” and “Poor communication behavior (8–16 points).” Patient Trust was categorized as “Full trust (40–50 points),” “Partial trust (21–39 points),” and “Lack of trust (10–20 points).” Patient satisfaction was categorized as “Full satisfaction” (16–20 points), “Partial satisfaction” (9–15 points), “No satisfaction” (4–8 points). For age, the cutoff was taken at 50 years of age as this was 1 standard deviation from the mean age. For waiting time as well, it was grouped into ≤1 h and more than 1 h (inclusive of waiting time of >1 h, 1–2 h, and >3 h). As the facilities in the study are aiming to limit their maximum waiting time of 1 h, the cutoff waiting time was taken at 1 h.

Analysis was done using Spearman's Correlation, chi-square tests and binary univariate and multivariate logistic regression using 0.05 significance level to check for any association between the independent and dependent variables. The results of the simple logistic regression analysis were presented as beta, crude odds ratio at 95% confidence interval (CI) and *p*-values. After the completion of the interview, all the audio recorded information was transcribed into a word document. Audio recording was heard three times and transcribed data was corrected accordingly. The final transcribed data were then analyzed using manual thematic analysis. Thematic analysis was guided by a 6 -phase guide framework by Braun and Clarke (35). The data were coded first and after reviewing this list, some codes were revised and added as well. These coded statements were then grouped together and from this, common themes were identified, prioritizing codes related to patient satisfaction and its determinants. Ethics approval was granted by the Fiji National University College Health Research Ethics Committee (CHREC) and from the Fiji National Research Ethics Review Committee (FNRERC). Participants were only recruited upon written consent was obtained.

## Results

**Figure 1:**
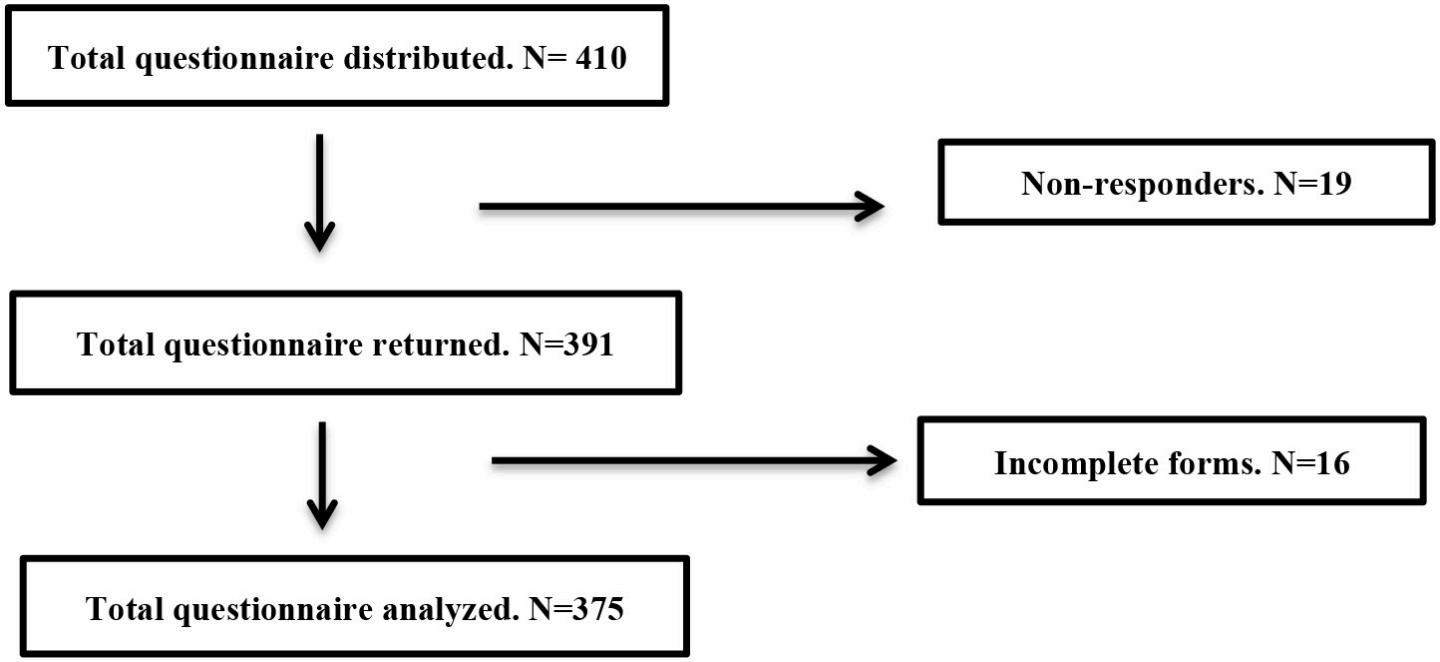
Flow of questionnaires distributed.

As summarized in Figure 1, from the total of 410 questionnaires were distributed, 375 completed forms were analyzed, 16 incomplete forms, and 19 forms were not returned. From the 375 questionnaires, there were 5 Fijian version questionnaire, 3 Indian version questionnaire and the rest was in English.

Table 1 summarized the characteristics of the participants. The mean age was 38 years (SD = ±15). Majority participants were female (229, 61.1%) and of i-Taukei (206, 54.9%) ethnicity. These figures are similar to the national ethnic distribution; 56.8% iTaukei, 37.5% Indo-Fijian, and 5.7% other ethnic groups. Most of the participants were educated and studied till either secondary or tertiary level (343, 91.5%). More than half the participants were employed (216, 57.6%) while 13.3% (50) were studying, 9.9% (37) were unemployed and 19.2% (72) were doing domestic duties. For most of the participants, it was more than 3rd visit to the health center (259, 69.1%) while for 10.4% (36) of the participants it was their first visit. As for the waiting time, majority of the patients (70%) had to wait more than an hour before seeing the doctor while 30% of them got seen within 1 h.

**Table 1:** Characteristics of participants (*n* = 375).

**Characteristic**	**No. of participants (*n*)**	**Percentage (%)**
**Age (mean ± SD)**	38 ± 15 years	
18–50 years	293	78.1
>50 years	82	21.9
**Gender**		
Male	146	38.9
Female	229	61.1
**Ethnicity**		
i-Taukei	206	54.9
Indo-Fijian	141	37.6
Others	28	7.5
**Education level**		
Higher	190	50.7
Secondary	153	40.8
Lower	32	8.5
**Employment status**		
Studying	50	13.3
Working	216	57.6
Unemployed	37	9.9
Domestic duties	72	19.2
**No. of visits to health center**		
>3 visits	259	69
2nd visit	77	21
1st visit	39	10
**Waiting time**		
>1 h	263	70
≤1 h	112	30

As seen in Table 2, the mean score for doctors' communication behavior was 30 points (±5) with minimum score of 16 points and maximum score of 40 points. Majority of the participants perceived doctors' communication behavior as fair (53%), followed by good (45.6%) and poor (0.8%).

**Table 2:** Distribution of participants based on levels of communication, trust, and patient satisfaction.

	**Points scale**	***N* (%)**	**Mean (±SD)**
**Communication behavior**			30 ± 5
Good	32–40	171 (45.6)	
Fair	17–31	201 (53.6)	
Poor	8–16	3 (0.8)	
**Trust**			38 ± 5
Full trust	40–50	144 (38.4)	
Partial trust	21–39	229 (61.1)	
Lack of trust	10–20	2 (0.5)	
**Patient satisfaction**			16 ± 3
Full satisfaction	16–20	260 (69.3)	
Partial satisfaction	9–15	106 (28.3)	
Dissatisfied	4–8	9 (2.4)	

The mean score for patient trust was 38 points (±5). The minimum score was 20 points with highest score of 50 points. Majority of the participants had partial trust in their doctors (61.1%), followed by full trust (38.4%) and lack of trust (0.5%). The mean score for patient satisfaction with their consultation was 16 points (±3). The minimum score was 5 points with maximum score of 20 points. More than two thirds (69.3%) of the participants were fully satisfied with the consultation, 28.3% were partially satisfied, and 2.4% were not satisfied with their consultation.

There were 4 statements used to assess patient satisfaction. As Table 3 reveals, majority of the participants either strongly agreed or agreed that they were totally satisfied with their consultation with the doctor (80.6%), satisfied with the treatment given to them (81.6%), that the staff were friendly and approachable (79.5%), and that they would prefer to come back to this doctor if they get sick again (80.5%).

**Table 3:** Participants response to patient satisfaction related statements.

	**Strongly agree and agree—*N* (%)**	**Not sure *N* (%)**	**Strongly disagree and disagree—*N* (%)**
I am totally satisfied with my consultation with the doctor.	302 (80.6)	47 (12.5)	26 (6.9)
I am satisfied with the treatment which I was given.	306 (81.6)	37 (9.9)	32 (8.5)
The staffs were friendly and approachable.	298 (79.5)	41 (10.9)	36 (9.6)
I would prefer to come back to this doctor if I ever get sick again.	302 (80.5)	44 (11.7)	29 (7.8)

For patient satisfaction, 84.7% of those over 50 years were fully satisfied with their consultation as summarized in Table 4. Almost three quarters of females had full satisfaction with consultation, while 62.3% of males were fully satisfied with the consultation. Close to three quarters of Indo-Fijians were fully satisfied with their consultation, while 67.9% of i-Taukei and 60.7% of Others were fully satisfied with their consultation. For education, those with lower level of education, 84.3% of them were fully satisfied. Eighty-four-point seven percent of those doing domestic duties were also fully satisfied. Of those who visited the health center more than 3 times, 72.2% of them were fully satisfied while 61% of those who came a second time and 66.7% of those who came the first time were fully satisfied. Almost 90% of those who waited <1 h were fully satisfied while those who waited more than 1 h, 62.7%, were fully satisfied. As for those who perceived doctor's communication behavior as good, 89.5% of them were fully satisfied with the consultation while those who perceived it as either fair or poor, 52.5 % of them were fully satisfied. Those who had full trust in their doctors, 95% were fully satisfied with the consultation where as those who had partial or lack of trust in their doctors, 53.3% were fully satisfied with their consultation.

**Table 4:** Level of patient satisfaction amongst participants based on independent variables.

**Independent variable**	**Full patient satisfaction (%)**	**Partial/no patient satisfaction (%)**
Overall	260 (69.3)	115 (30.7)
**Age**		
18–50 years	193 (65.9)	100 (34.1)
>50 years	67 (84.7)	15 (15.3)
**Gender**		
Male	91 (62.3)	55 (37.7)
Female	169 (73.8)	60 (26.2)
**Ethnicity**		
i -Taukei	140 (67.9)	66 (32.1)
Indo-Fijian	103 (73)	38 (27)
Others	17 (60.7)	11 (38.3)
**Education level**		
Higher	124 (65.3)	66 (34.7)
Secondary	109 (71.2)	44 (28.8)
Lower	27 (84.3)	5 (15.7)
**Employment status**		
Studying	30 (60)	20 (40)
Working	141 (65.3)	75 (34.7)
Unemployed	26 (70.3)	9 (24.3)
Domestic duties	61 (84.7)	11 (15.3)
**No. of visits to HC**		
>3 visits	187 (72.2)	72 (27.8)
2nd visit	47 (61)	30 (39)
1st visit	26 (66.7)	13 (33.7)
**Waiting time**		
≤1 h	95 (84.8)	17 (15.2)
>1 h	165 (62.7)	98 (37.3)
**Communication behavior**		
Good	153 (89.5)	18 (10.5)
Fair/poor	107 (52.5)	97 (47.5)
**Patient trust**		
Full	137 (95.1)	7 (4.9)
Partial/lack of	123 (53.3)	108 (46.7)

For inferential analysis, the univariate logistic regression findings are summarized in Table 5. Age, gender, employment status, education level, waiting time, doctors' communication behavior and level of patient trust had significant association with patient satisfaction at 0.05 level of significance independently. Those over 50 years, females, those doing domestic duties, those with lower level of education, those who got seen within 1 h, those who perceived doctors' communication behavior as good and those who had full trust in their doctors, they were more likely to be fully satisfied with their consultation.

**Table 5:** Univariate analysis to find determinants of patient satisfaction.

**Variable**	**Coefficient (β)**	**OR**	***P-*value**	**95% CI**
**Age**				
^*^18–50 years	0			
>50 years	0.839	2.30	^**^0.007	1.258–4.258
**Gender**				
^*^Female	0			
Male	−0.532	0.587	^**^0.019	0.376–0.917
**Ethnicity**				
^*^i-Taukei	0			
Indo-Fijian	0.254	1.278	0.310	0.796–2.051
Others	−0.317	0.729	0.445	0.323–1.643
**Employment status**				
^*^Working	0			
Unemployed	0.504	1.655	0.218	0.742–3.689
Studying	−0.226	0.798	0.483	0.424–1.500
Domestic duties	1.082	2.950	^**^0.02	1.464–5.943
**Education level**				
^*^Lower level	0			
Secondary level	−0.779	0.459	0.133	0.166–1.268
Higher level	−1.056	0.348	^**^0.038	0.128–0.946
**No. of visits to HC**				
^*^>3 visits	0			
2nd visit	−0.505	0.603	0.063	0.354–1.027
1st visit	−0.261	0.770	0.470	0.375–1.581
**Waiting time**				
^*^>1 h	0			
<1 h	1.20	3.319	^**^0.0001	0.870–5.889
**Communication behavior**				
^*^Fair/poor	0			
Good	2.042	7.706	^**^0.0001	4.400–13.495
**Patient trust**				
Partial/Lack				
Full	2.844	17.18	^**^0.0001	7.704 – 38.332

* *Reference category*.

** *Significant result at 0.05 level of significance*.

Stepwise backward selection was used to get the final logistic regression, as summarized in Table 6. With multicollinearity check, there was moderate correlation between communication behavior and patient trust (*r* = 0.67), hence communication behavior was also removed from the logistic regression. After controlling the variables, only gender, number of visits, waiting time and level of patient trust had significant association with patient satisfaction at 0.05 level of significance. While males and those coming to the health center for first time were less satisfied with their consultation, those who got seen with 1 h and those who had full trust in their doctors were more likely to be satisfied with their consultation.

**Table 6:** Multivariate binary analysis to find determinants of patient satisfaction—final logistic regression.

**Variable**	**Coefficient (β)**	**Adjusted OR**	***P-*value**	**95% CI**
**Gender**				
^*^Female	0			
Male	−0.785	0.456	^**^0.004	0.269–0.775
**No. of visits to health center**				
^*^>3 visits	0			
2nd visit	−0.662	0.419	0.570	0.171–1.027
1st visit	−0.870	0.516	^**^0.04	0.275– 0.969
**Waiting time**				
^*^>1 h	0			
<1 h	1.71	3.305	^**^0.0001	1.685–6.174
**Trust level**				
^*^Partial/lack of	0			
Full	2.928	18.68	^**^0.0001	8.131–42.938

* *Reference category*.

** *Significant result at 0.05 level of significance*.

This logistic regression equation had Nagelkerke R square of 0.368, hence explained 36.8% of the variance in the data. Hosmer and Lemeshow test (chi-square = 9.4, *p* = 0.224) tells us that this model fit well. The ROC curve area was 0.806 and this tells us that this model was accurate in predicting patient satisfaction.

The thematic analysis process that was applied to the transcribed data to show the key concepts that were evident in the data. Similar findings were noted from this qualitative approach. Themes identified from the data included “Personality of the Doctor,” “Treating the patient as an individual,” “Understanding your sickness,” and “Is it worth waiting.”

### Personality of the Doctor

When talking about their consultation encounter, the doctors' communication behavior was mentioned in either a positive or negative way together with the doctor's attitude and how the doctors treated them. During their consultation, for majority patients, the attitude of the doctors did carry weight. When the doctors were nice to the them, talked in normal tone, their politeness and not sounding harsh or telling off patients, the patients were happier with their consultation. “*The doctor was friendly and polite”* (49 years, i-Taukei male). While another 59 years old i-Taukei male said, “*I feel comfortable, they are not harsh or rough with you… they do their part.”* Some patients appreciated the way they were welcomed by the doctors and their tone when they talk. “*The way doctors' approach me…uhm and the way they talk and their tone matters”* (23 years, Indo-Fijian male). “*Their first expression… the way they look and greet…I feel happy”* (42 years, i-Taukei male). One patient even felt that some doctors go out of their way to make them feel comfortable during the consultation. “*I'll say probably he had treated me as a person… he left his level of being a doctor and explained to me as a lay person, cared for me… I was comfortable”* (30 years, Indo-Fijian male). While majority had appreciated the doctor's attitude, few experienced the opposite from their doctors which lead to dissatisfaction with their consultation. The doctor was rude to them and did not talk or explain nicely to them.

“*…the doctor was rude, just saw, wrote the prescription… just sitting and didn't ask”* (38 years, Indo-Fijian female). Patients felt frustrated and dissatisfied when they encounter an arrogant doctor. Those who didn't interact much with the patients and personality stands out as stubborn, were not liked or appreciated by the patients. “*Felt angry as doctor didn't explain or examine… couldn't ask because of fear… looked bit stubborn doctor”* (39 years, Indo-Fijian female).

### Treating the Patient as an Individual

Majority patients appreciated the fact when the doctors treated them as an individual person rather than some patient with a complain. When doctor put in effort to engage patients into conversation with an attempt to understand the patient, patients seemed to be more satisfied with their consultation. The doctors listened and understood what the patients concern was regarding their health. One of the participants, 48 years old Indo-Fijian male stated, “*It was a good experience with doctors…I trust the doctor… the way they talk is good, I could ask them, the doctors and nurses are asking things and cared about my need and explained.”* Patients indirectly highlighted the importance of communication and trust when it comes to understanding the patients. “*I trust the doctors because of how they have treated me…. Doctor says morning to me. The face was welcoming, and I feel free to talk to the doctor”* (46 years, i-Taukei female).

Patients showed importance to the fact that doctors involve them in the conversation and in the management plan and let patients ask questions as well. “*They respect your opinion… they gave me time to ask the questions and I asked them more questions”* (51 years, i-Taukei female). While another participant, 51 years old, i-Taukei female stated, “*Yes, they discuss the treatment with me, explained the side effects and asked if I was allergic to any medicine.”* Some patients mentioned that they were not involved during the consultation process but would have appreciated if it was done. Sometimes it is difficult for patients to share all information with the nurse which they would rather share with the doctors. “*…most questions have been asked by the nurse, not by doctor. Nurse just gives, and doctor reads it. She can read and ask again, it's a follow up questions… sometimes you can say different thing to nurse”* as stated by 43 years, i-Taukei male.

In contrast, when patients were not involved in the treatment plan or their views and opinions were not considered by the doctors, they were some wort disappointed. “*I asked them to give me the medicine from another IV site, but they didn't listen at XXX (name of HC deleted) …my hand got swollen”* (48 years, Indo-Fijian male). Also, in-terms of understanding the patient in context of their financial state, few patients showed disappointment as they were not informed about the medicine and the doctor didn't care to inform them that they had to buy the medicine, as some patients might not afford to buy the medicine. Thirty years old i-Taukei male stated, “*The medicine was not here, and I have to buy the medicine. He (the doctor) never ask and told that I need to buy it. I would appreciate if the doctor can ask.”*

### Understanding Your Sickness

Most of the time patients have lot of questions in their mind concerning their sickness and health and only when all this questions and doubts are answered and cleared, then only they have the total satisfaction with their consultation. This was evident from statement by a participant, 59 years i-Taukei male, “*The 100% is how they perform when they explain the sickness to you, the right medication and what it can do and the side effects.”* Just with proper communication and explaining things well to the patient, it gets rid of their anxiety and frustration. “*In the beginning, I am bit scared but when I enter the room and the way the doctor greets me, I can understand that I can be comfortable with this doctor”* (31 years, Indo-Fijian male).

Together with communication, trust was also linked to them being satisfied with the explanation and consultation. Knowing that medical information is readily available over internet, it can sometimes be a challenge to explain things to patients when they come in with incomplete information on a particular issue in their mind. As highlighted from by this thematic analysis, if the doctor communicates effectively with their patients and are able to gain patients trust, patients are convinced with the explanation provided. A 42 years old Indo-Fijian male stated, “*…. the way they saw me and explained me and of course they are doctors, I trust that person. My doctor saw me well…”* (42 years, Indo-Fijian male).

For those who were expecting the doctor to provide them more information concerning their health, they showed disappointment when they were not provided with all the information. “*…but at times might not be all the information that I needed. Sometimes the doctors don't explain well, depends on the doctors…”* (66 years, Indo-Fijian male). Also, as mentioned, when the doctors don't engage patients into conversation and provided little or no information concerning their sickness, those patients were neither satisfied with their consultation nor trusted the doctor was giving them the right management. “*…the doctor was rude, just saw, wrote the prescription… just sitting and didn't ask anything…I was also afraid to ask. I am not sure whether I should take the medicine or not”* (38 years, Indo-Fijian female).

### Is It Worth Waiting?

Participants did point out the fact that the waiting time was long, and they would appreciate if it would be lesser. For majority patients, they would be more satisfied if they had a good consultation and shorter waiting time. Though when asked as to whether they would prefer a shorter waiting time with shorter incomplete consultation or a proper consultation despite waiting bit longer, participants proper consultation was more important than a shorter waiting time. “*…. Patient get sick with long waiting time…. I would prefer better consultation then shorter waiting time”* (23 years, Indo-Fijian male). Participants were more satisfied when then had a good consultation despite the long wait. A 35 years old i-Taukei male stated, “*If the consultation is good, then it is worth waiting…”* It was also noted that for some, the long waiting time was a routine, and they did not expect anything better. “…*No, the waiting time does not affect me, if your mindset is ok, you can see the big crowd here…you have to be patience”* as stated by a 59 years old i-Taukei Male.

## Discussion

This study's finding revealed that for a PIC like Fiji, age, gender, education level, waiting time, doctors' communication behavior and patient trust level were significantly associated with patient satisfaction independently. It was also noted that doctors' communication behavior positively affected patient trust as well. After adjusting for all the variables, gender of patient, number of visits to the health center, waiting time and patient trust were significantly associated with patient satisfaction. From all these factors, patient trust and doctors' communication behavior and waiting time were more strongly associated with patient satisfaction independently. Like Jaipul and Rosenthal's study finding which showed that those older age groups till 60 years were more satisfied with their consultation than the younger population, this study also showed the same (8). This may be due to the fact that this age group have less expectation from their doctors. Also, from their younger times, there might have been an improvement in the health services overall, thus the older age group may be satisfied due to the changes compared to their past encounters. This was evident from the qualitative study when older patients talked about their experience and the changes noted in the doctor behavior toward them.

Females were more likely to be fully satisfied with their consultation than males This finding differed from the findings of a study carried out by Weisman et al. which showed that there were no major gender differences in satisfaction (37). Again, findings of another study done by Schmittdiel et al. showed that overall satisfaction ratings were similar for female and male patients, though, their study did reveal differences in patient satisfaction related to the gender of the patient and of the physician (36). This could be one of the reasons behind the gender difference from this study; the gender of the doctors who consulted them. From the qualitative finding, there was no major difference noted in the gender and satisfaction; there were similar positive and negative reactions from both males and females. Few studies have shown that females visit doctors more often than males (7, 8). This may be explained by continuity of care which is linked to patient satisfaction (12).

Those who visited the HC more than three times were more likely to be fully satisfied than those who came for the first time. A study conducted in South Korea did show that frequency of visit did have a significant effect on patient satisfaction (10). Though their findings revealed that those coming for the 2nd to 6th visit were less likely to be satisfied compared to the 1st visit or more than 6th visit in contrast to the findings of this study. Bleustein et al. showed in their study that those receiving care for the first time at a particular provider were less satisfied than their counterparts who have previously received care there (11). A qualitative study that was conducted in Denmark revealed that the relationship between the doctors and the patient may be reinforced by greater continuity and recognition (12, 38). Thus, the findings of this study might be partially attributed to continuity of care with frequent visits.

This study also showed that those who waited less than an hour, were more likely to be fully satisfied than those who had to wait longer than 1 h. This finding was similar to previous studies done (11, 39, 40). Bleustein et al. in their study, also showed that scores for patient satisfaction was negatively correlated to waiting time (11). Similarly, Kreitz et al. showed that as waiting time increases, patient satisfaction decreases (39). Though Anderson et al. also showed that longer wait time affected patient satisfaction negatively, the strongest predictor was the time spent with the physician rather than waiting time (40). The qualitative findings of this study also showed despite patients voicing out the long waiting time, they were satisfied with proper consultation. Similar to Anderson et al study, in this study, patient's trust in the doctor was the strongest predictor of patient satisfaction followed by waiting time.

Those who had full trust in doctors were more likely to be fully satisfied with their consultation. This finding is similar to previous studies done (14, 41–43). A study conducted by Lan et al. in Taiwan showed that together with interaction and empathy, trust was positively correlated with patient satisfaction (13). Chang et al. in their study also showed that perception of trust among patients positively influenced their satisfaction (14). Another study conducted in China by Shan et al. revealed that patient satisfaction was positively associated with a higher level of trust and that from all the predictors of patient satisfaction, trust was the strongest predictor (42). The odds of having full patient satisfaction for those with full trust in their patients was similar to this study, in terms of being relatively high. The qualitative findings of this study also highlighted that when doctors are able to connect with their patients with proper communications, patients turn to trust their doctors and the treatment provided. This ended up in patients being satisfied with their consultation and treatment.

This may be due to the fact that those who had full trust also had doctors with good communication behavior, hence the patients are likely to have understood about their health problem and management would have been in accord, resulting in a high chance of satisfaction. A study conducted by Platonova et al. also showed that patient trust and good interpersonal relationships with the primary care physician are major predictors of patient satisfaction (43). Interpersonal relationship is inclusive of the communication behavior of doctors. In this study, communication behavior was a strong predictor of patient trust and patient trust was a strong predictor for patient satisfaction, thus trust acts as an interlink between doctors' communication behavior and patient satisfaction. Some studies have shown correlation between communication skills of the doctors (15, 16).

Clever et al. conducted a study to find the relationship between physician's communication behavior and patients' overall satisfaction with hospital care showed a significant positive relationship between overall ratings of attendings' communication behaviors and overall satisfaction (15). Similarly, Biglu et al. carried out a descriptive study Iran which showed strong correlation between communication skills of doctors and patient satisfaction (16). The qualitative findings from this study showed that when doctors communicate to their patients regarding their sickness and the management plan, the patients are more satisfied with consultation. As noted, determinants of patient satisfaction in PICs, namely, patient trust, doctor-patient communication and waiting time is similar to determinants of patient satisfaction globally, though other factors (gender and no.of visists) varied upon countries.

## Limitations

The present review has several limitations. Though, the patients were selected using random sampling, they were only selected upon their will. Hence, this would have introduced self-selection bias. There might have been a possibility of acquiescence bias as the qualitative questionnaire used both negative and positive statements, as evident from a few of the completed forms. Though, the name of the participants was not revealed, the participants did fill in their name in the questionnaire. Hence, there is a possibility of social desirability bias as well. The quantitative questionnaire did not capture the financial status of the participants and the gender of the doctors and this might have affected the results slightly as studies have shown that socioeconomic status affects patient trust and satisfaction (44, 45). The reliability of the structured questionnaire was not done before being used, though it was calculated using this study sample.

## Summary and Conclusion

Building better trust with proper communication helps in improving patient satisfaction with the consultation and treatment provided and this in turn improves treatment adherence and better health outcomes. This study has shown that currently two thirds of the Suva sub-divisional health center utilizers are fully satisfied with their consultation. There are several factors associated with patient satisfaction individually, such as age, gender, education level, number of visits, waiting time, communication behavior, and interpersonal skills of doctors and patient trust. In concordance with other countries globally, patient trust, waiting time, and communication behavior and interpersonal skills of the doctors had similar relation to patient satisfaction in PIC, Fiji. Some of these are modifiable, such as communication, trust and waiting time, while some are not. Therefore, it would be recommended for health service managers to upgrade their staffs' communication skills and come up with strategies to lessen the waiting time. This study suggest that's patient satisfaction can be used as an indirect measure of health outcomes either subjectively or objectively, though, it would be recommended for further studies to be carried out to better understand the link between patient satisfaction and health outcomes with direct measurement of clinical health outcomes.

## Ethics Statement

Ethics approval was granted by the Fiji National University College Health Research Ethics Committee (CHREC) and from the Fiji National Research Ethics Review Committee (FNRERC). Participants were only recruited upon written consent was obtained.

## Author Contributions

All authors took part in the design of the study. SC drafted the manuscript with input from all authors. Research proposal was guided by MM and PW. The data was collected and analyzed by SC and revised by MM and PW. All authors participated in the preparation and approved the final manuscript for publication.

### Conflict of Interest Statement

The authors declare that the research was conducted in the absence of any commercial or financial relationships that could be construed as a potential conflict of interest.
